# A nap before retrieval reduces false identifications in target absent lineups

**DOI:** 10.1038/s41598-025-20471-2

**Published:** 2025-10-17

**Authors:** Matías Bonilla, Cristian García Bauza, Cecilia Forcato

**Affiliations:** 1 Laboratorio de Sueño y Memoria, Departamento de Ciencias de la Vida, Instituto Tecnológico de Buenos Aires (ITBA), Iguazú 341 (1437) Capital Federal Buenos Aires, Buenos Aires, Argentina; 2 Consejo Nacional de Investigaciones Científicas y Tecnológicas (CONICET), Buenos Aires, Argentina; 3 PLADEMA, Universidad Nacional del Centro, Tandil, Buenos Aires Argentina

**Keywords:** Human behaviour, Cognitive neuroscience, Learning and memory

## Abstract

Eyewitness misidentifications are a major cause of wrongful convictions. While most research has focused on the role of sleep in memory encoding and consolidation, recent findings suggest that non-REM sleep may also enhance retrieval processes by restoring executive and monitoring functions. Here, we investigated whether a short nap before retrieval could improve performance in forensic memory tasks. Participants watched an aversive video of a robbery and, after 24 h, either took a 60-minute nap (Sleep group) or stayed awake (Wake group) before completing a series of memory tasks, including face recognition (target-present and target-absent lineups), free recall, context recognition, and temporal order. A nap prior to retrieval selectively reduced false identifications in target-absent lineups, increasing correct rejections without affecting performance in target-present trials. Temporal order memory also improved in the Sleep group, whereas no significant differences were observed in free recall or context recognition. Confidence-accuracy calibration was stronger in the Sleep group, particularly when the perpetrator was absent. These findings suggest that a brief nap prior to memory retrieval may improve the accuracy of eyewitness decisions by enhancing metacognitive monitoring and temporal discrimination, offering a simple, non-invasive intervention to reduce errors in forensic settings.

**Supplementary Information:**

The online version contains supplementary material available at 10.1038/s41598-025-20471-2.

## Introduction

Eyewitness memory plays a crucial role in legal investigations and courtroom decisions, often serving as the primary or even sole source of evidence when other forms are unavailable^1^. However, this reliance can be problematic. According to the Innocence Project, nearly 70% of wrongful convictions in the United States are due to eyewitness misidentifications, highlighting the fallibility of memory in forensic contexts^2^. Improving the accuracy and reliability of eyewitness testimony is therefore a critical goal for both scientific research and the legal system.

In the field of eyewitness memory research, two main types of factors have been identified as influencing memory performance: system variables and estimator variables. System variables refer to those factors that are under the control of the legal system and can be manipulated to improve the accuracy of eyewitness testimony^3^. These include aspects like lineup procedures (e.g., simultaneous vs. sequential lineups), instructions given to witnesses (e.g., informing them that the perpetrator may not be present), interview techniques (e.g., the cognitive interview method), and the way feedback is provided (e.g., avoiding confirming feedback after an identification)^4^. Estimator variables are factors that affect memory accuracy but cannot be controlled by the justice system^5^. Some examples of these variables include distance from the event^6^, alcohol intoxication^7^, exposure duration^8^, stress^9^, presence of a weapon (weapon focus effect)^10^, and own-race bias^11^. More recently, sleep has been proposed as another important estimator variable that may influence memory performance in forensic settings^12–14^.

Sleep is a biologically essential, homeostatically and circadian regulated process that restores and optimizes brain function^15^. Humans typically sleep in 90-minute cycles encompassing both rapid eye movement (REM) and non-REM sleep. Non-REM sleep, particularly slow-wave sleep (SWS), is characterized by increased slow wave activity (SWA, 0.5–4 Hz; slow oscillations 0.5–1 Hz plus delta 1–4 Hz), which according to the synaptic homeostasis hypothesis^16,17^, promotes the downscaling of synaptic strength built up during wakefulness. This synaptic renormalization restores neural efficiency, thereby enhancing the consolidation of previously acquired information as well as the encoding of new information upon waking. Importantly, naps of moderate duration (around 40 min of total sleep time) are likely to include Stage 2 and SWS while avoiding the onset of REM sleep, which typically occurs after ~ 70–90 min in healthy young adults^18^. This makes them particularly suitable for targeting the benefits of NREM sleep rich in SWA without introducing the additional effects of REM sleep.

Recent studies have begun to investigate the impact of sleep on eyewitness memory performance. Stepan et al.^13^ showed that sleep contributes to the enhancement of eyewitness performance. That is, sleep after encoding reduced false identifications in target-absent lineups, potentially by increasing conservative response tendencies. However, Morgan et al.^14^ did not replicate these effects, leaving open questions about the conditions under which sleep benefits eyewitness accuracy. To date, no research has specifically examined the impact of sleep on memory retrieval per se, independent from its effects on consolidation and encoding. The present study was designed to address this gap by testing whether a short nap taken just before a retrieval task could optimize eyewitness performance, potentially through NREM sleep-related restoration of neural efficiency.

Prefrontal and temporal areas are involved in executive processes necessary for accurate memory-based decisions^19,20^. Sleep deprivation impairs cognitive performance on various tasks involving inhibitory control, task switching, working memory and self-monitoring processes^21^. Although estimator variables generally show little variability in confidence-accuracy relationships^5,22–25^, mixed results have been found under sleep deprivation condition. While Blagrove and Akehurst^26^ reported impairments in confidence-accuracy calibration (correspondence between expressed confidence and actual accuracy) under sleep loss, Baranski et al.^27^ found no such effect after 28 h of wakefulness.

Beside system and estimator variables, confidence is an important component of eyewitness testimony that can ultimately determine the outcome of a trial. Although it was long considered an unreliable indicator of accuracy, recent research shows that, under optimal conditions, high-confidence identifications are highly accurate^28^. Tools such as Confidence-Accuracy Characteristic (CAC) curves have been developed to quantify this relationship^14^. Confidence judgments are supported by regions such as the orbitofrontal and prefrontal cortex, which contribute to self-monitoring, self-evaluation, and executive control^29^. Thus, confidence can reflect memory accuracy in a lineup if conditions are pristine^28^, but it also depends on individuals’ capacity to monitor and evaluate the strength and perceived reliability of their retrieved memories^30^. Thus, we propose that sleep not only influences memory accuracy but also could impact confidence judgments in eyewitness memory.

Finally, other memory processes are highly relevant in forensic settings, where witnesses are often asked not only to identify a suspect but also to recall details of the crime scene and properly reconstruct the sequence of events. Context recognition tasks assess the ability to distinguish between true and altered environmental features, a capacity linked to hippocampal binding and reinstatement processes that support remembering where and when events occurred^31,32^. Such contextual information is frequently critical in legal testimonies, for example when establishing the presence of specific objects, locations, or circumstances surrounding a crime. Similarly, temporal order judgments measure the ability to sequence events in time, a central aspect of episodic memory^33^ that relies on frontal and posterior brain regions^34^. In forensic contexts, errors in sequencing can distort the perceived narrative of a crime and contribute to false memories or source misattributions, such as recalling a detail from the wrong event or attributing it to the wrong individual, which can have serious legal consequences^1,35^. Both tasks also involve monitoring and executive functions^36^, which are vulnerable to sleep deprivation and may benefit from the restorative role of NREM sleep^21^.

It is worth noting that a key translational goal in eyewitness memory research is to bridge science and the legal system by developing practical tools to reduce wrongful convictions. Interventions targeting encoding are not feasible, as crimes occur unexpectedly and beyond the witness’s control. Likewise, opportunities to influence consolidation are limited, because witnesses may not report the event immediately and post-event sleep often cannot be arranged. In contrast, retrieval occurs at predictable, scheduled points in the judicial process, such as during police lineups, when the environment and the witness’s state can be more easily controlled. This makes retrieval a realistic and actionable target for interventions aimed at improving eyewitness accuracy. Based on this rationale, we investigated whether a brief nap before memory retrieval could enhance eyewitness performance, particularly by reducing false identifications. We also explored potential effects on temporal order judgment and free recall as secondary outcomes, given their relevance for episodic integration and executive monitoring in forensic contexts.

## Results

**Fig. 1:**
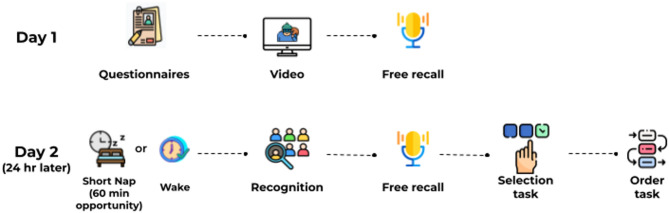
Experimental procedure. The procedure was divided into two parts: First, on day 1, the participants completed the mood scales and sleep quality index. Then, they watched the aversive video and finally made a free recall of it (short-term testing). 24 h later (day 2) participants went through the last session. First, they made the recognition task and after that, they made the free recall (long-term testing). At last participants completed the selection and chronological order task and finished the study. Icons taken from Freepik: https://freepik.es.

To examine the impact of a short nap on retrieval of eyewitness memory, participants watched a video of a robbery on day 1, and on day 2 they were given the opportunity to take a nap of up to 60 min or stayed awake. After this period, they were tested on facial recognition (Target-present lineups (TP) and Target-absent lineups (TA)), free recall, context recognition, and temporal order memory (Fig. 1).

### Facial recognition task

In the TP lineups, both groups identified the perpetrator at above chance-levels [chance = 14.28% (lineup divided by the 7 options) at both conditions, χ²_wake_(1,88) = 18.56, *p* < 0.001; χ²_sleep_(1,102) = 22.86, *p* < 0.001]. However, no significant association was found between group, condition and lineup performance (χ² (2,95) = 1.044, *p* = 0.593), suggesting that napping did not influence target identification when the perpetrator was present. By contrast, group performance differed significantly in the TA lineups. Both groups performed above chance level (χ²_wake_(1, 88) = 9.24, *p* = 0.002; χ²_sleep_(1, 102) = 25.80, *p* < 0.001; Fig. 3), but the Sleep group demonstrated a higher rate of correct rejections (56.86%) compared to the Wake group (31.82%) (Table 1). A chi-square analysis revealed a significant association between group and performance (χ²(1, 95) = 5.98, *p* = 0.014), indicating that the nap selectively improved rejection accuracy in the absence of the perpetrator (Table 1).

**Table 1:** Performance summary in the testing Session. Percentage of identification outcomes for the wake and sleep groups on the testing session (day 2) divided by condition (present and absent).

Target-Present condition			
Group	Hits	False alarms	Incorrect rejections
Wake (*N* = 44)	47.73% (*N* = 21)	36.36% (*N* = 16)	15.91% (*N* = 7)
Sleep (*N* = 51)	50.98% (*N* = 26)	27.45% (*N* = 14)	21.57% (*N* = 11)

Target-Absent condition			
Group	False alarms	Correct rejections	
Wake (*N* = 44)	68.18% (*N* = 30)	31.82% (*N* = 14)	
Sleep (*N* = 51)	43.14% (*N* = 22)	56.86% (*N* = 29)	

**Fig. 2:**
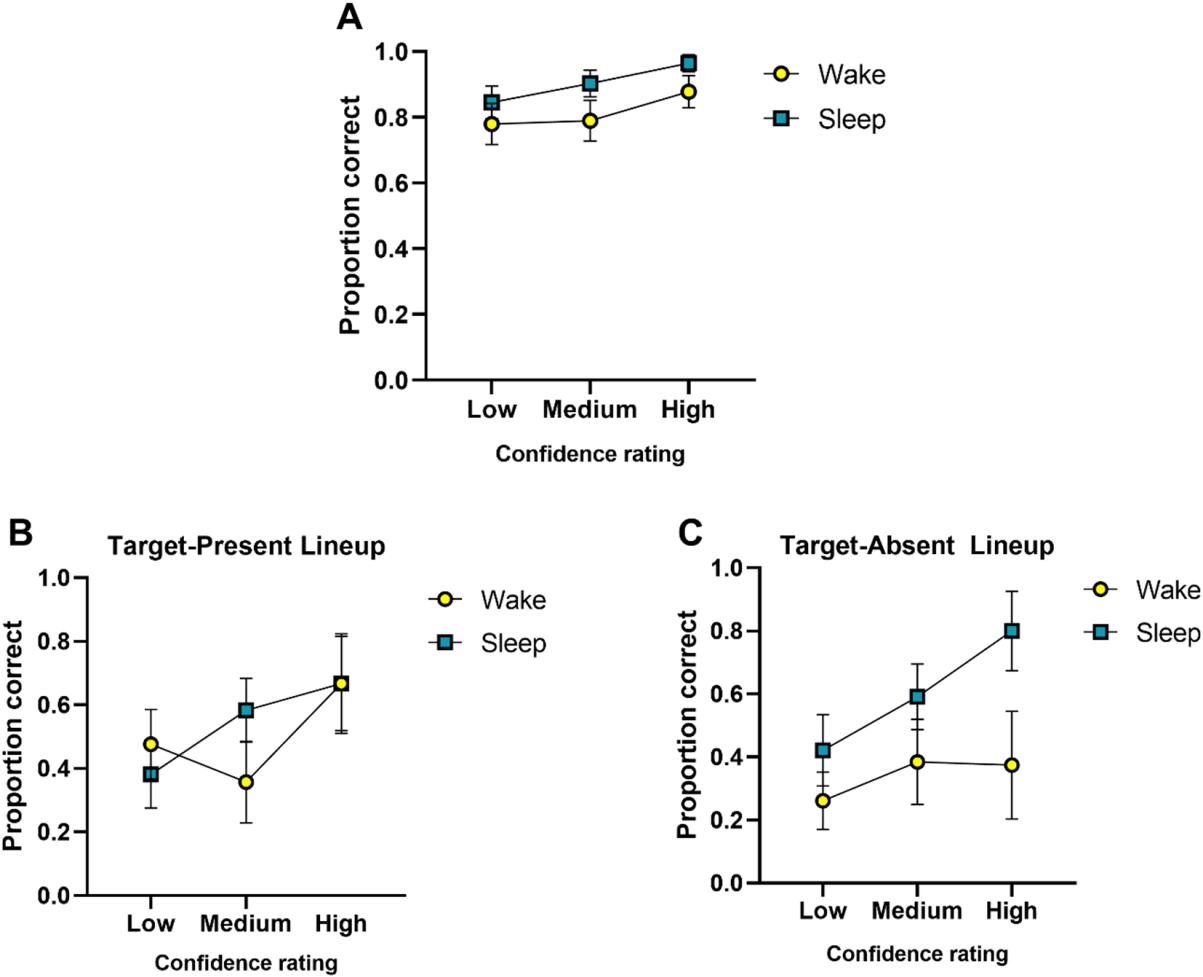
**(A)** Confidence-Accuracy Characteristic (CAC) curve showing the exponential relationship between confidence and recognition accuracy, collapsed across conditions. Error bars indicate the standard deviation (SD). **(B)** and **(C)** CAC curves separated by condition (target-present and target-absent) for each group (Sleep and Wake), illustrating how accuracy scales with confidence under each lineup context. Error bars indicate the standard deviation (SD).

Furthermore, the confidence-accuracy characteristic (CAC) analysis revealed that both groups exhibited similar performance, maintaining the expected exponential relationship between confidence and accuracy (Fig. 2A). However, the Sleep group consistently showed slightly higher accuracy across all confidence levels, suggesting a stronger confidence-accuracy relationship compared to the Wake group. When analyzing each lineup condition separately, several observations emerge. First, the Sleep group showed stable and high performance across both target present and absent lineups. In contrast, the Wake group performed better in the present lineup compared to the absent lineup (Fig. 2, B and C). This pattern is expected, as accuracy in the target-absent condition often declines due to the lack of a target. However, in this case, the Sleep group not only maintained but appeared to enhance its performance in the target-absent condition, suggesting a more robust confidence-accuracy relationship.

**Fig. 3:**
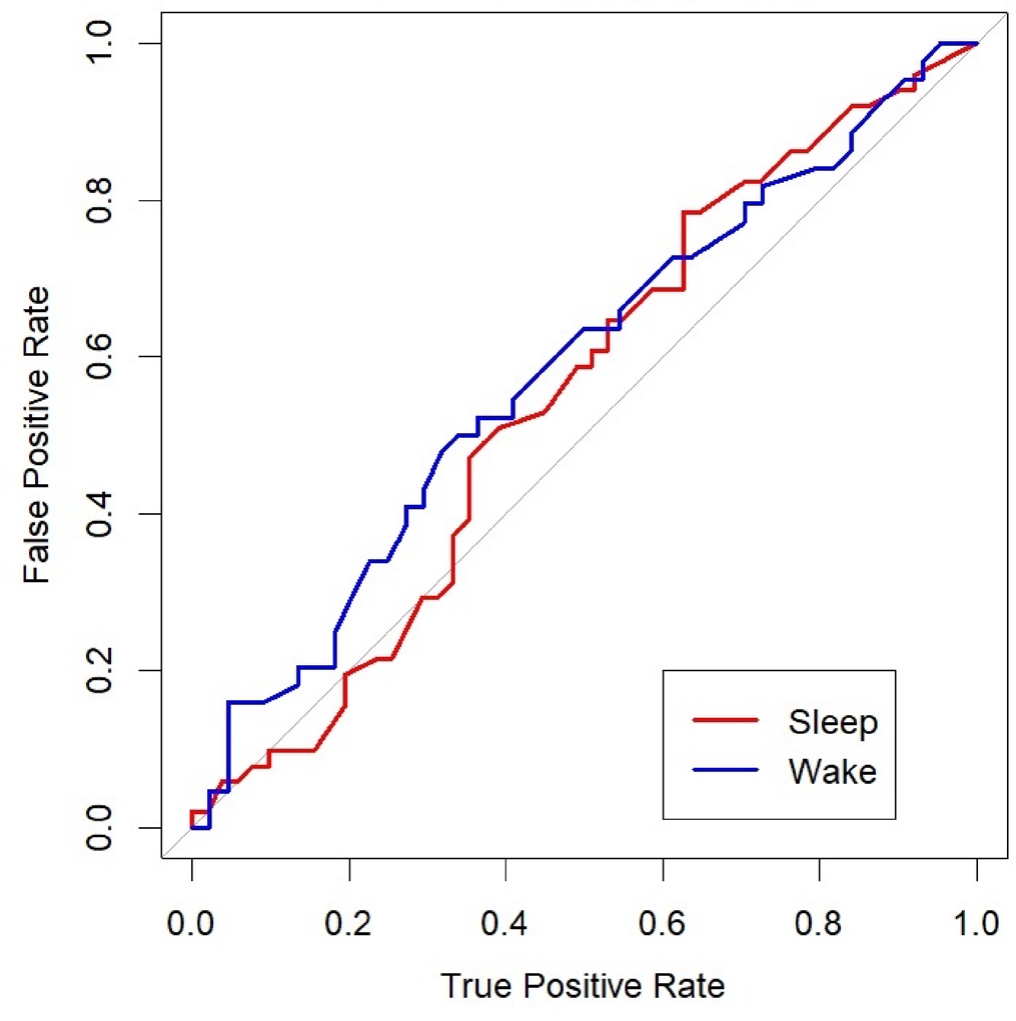
ROC curves. Receiver Operating Characteristic (ROC) curves for the Sleep and Wake groups, based on lineup decisions across both target present and absent conditions.

Regarding ROC curves, both groups showed AUCs close to chance level, with no significant differences between them (Fig. 3; Sleep group: AUC = 0.55, SD = 0.06; Wake group: AUC = 0.58, SD = 0.40; *p* = 0.69). This finding contrasts with the chi-square analysis, which revealed significant group differences in the Target-Absent lineup condition. Because standard ROC analysis integrates hit rates from TP lineups and false-identification rates from TA lineups into a single summary function (AUC), it collapses across conditions, so effects that arise only in one condition (e.g., a reduction in false identifications in TA lineups) may not be apparent in the overall AUC. To characterize these condition-specific patterns, we additionally report chi-square comparisons of response frequencies within TP and TA lineups and Confidence–Accuracy Characteristic (CAC) curves to summarize accuracy across confidence levels. These complementary analyses provide a descriptive view of accuracy and response tendencies by condition.

Taken together, these findings suggest that taking a nap prior to the lineup may significantly reduce false identification when the perpetrator is absent. By contrast, when the perpetrator is present, sleep does not appear to significantly influence recognition performance.

### Exploration of predictors of facial recognition performance

To better understand the factors influencing recognition performance, we conducted a series of logistic regression analyses with group (Sleep vs. Wake), condition (target-present [TP] vs. target-absent [TA]), and confidence level as predictors. For TP lineups, the dependent variable contrasted correct culprit identifications (coded as 1) versus filler identifications or incorrect rejections (coded as 0). For TA lineups, the dependent variable contrasted correct rejections (coded as 1) versus false identifications (calculated as the number of filler IDs divided by the nominal lineup size; coded as 0). This approach follows current eyewitness identification conventions when no designated innocent suspect is included in TA lineups (e.g., Mickes et al.^37^; Gronlund et al.^38^. The overall model was statistically significant (χ²(3) = 15.18, *p* = 0.002), indicating that the predictors improved model fit over the null model. Among the predictors, confidence emerged as a significant predictor of accurate decisions (B = 0.021, SE = 0.006, Wald(1) = 10.30, *p* = 0.001, Exp(B) = 1.021), suggesting that higher confidence was associated with increased odds of making the correct lineup decision. Neither group nor condition reached significance (B = 0.523, SE = 0.304, Wald(1) = 2.95, *p* = 0.086, Exp(B) = 1.686; B = −0.214, *p* = 0.480, respectively).

Since confidence was a significant predictor, we ran separate logistic regression models for TP and TA conditions. In TP lineups, the model was statistically significant (χ² = 11.29, df = 2, *p* = 0.004), with confidence significantly predicting correct culprit identifications (B = 0.034, SE = 0.011, Wald = 9.683, *p* = 0.002). Group was not a significant predictor (*p* = 0.939). In TA lineups, the overall model did not reach significance (χ² = 5.56, df = 2, *p* = 0.062), though confidence remained a significant predictor of correct rejections (B = 0.020, SE = 0.009, Wald = 5.258, *p* = 0.022). Group was again non-significant (*p* = 0.902). Finally, within the Sleep group, we examined whether sleep parameters predicted performance separately for TP and TA lineups. In TP lineups, confidence was the only significant predictor (B = 0.034, *p* = 0.032), with an odds ratio (OR) of 1.035, while %SWS and %S2 were not significant (B = 0.006, *p* = 0.715, B = 0.027, *p* = 0.070, respectively). In TA lineups, none of the predictors reached significance (confidence: B = 0.024, *p* = 0.077, OR = 1.024; %SWS: B = 0.000, *p* = 0.992; %S2: B = 0.006, *p* = 0.675). In summary, confidence consistently predicted lineup accuracy across conditions, while group and sleep architecture measures were not significant predictors.

### Free recall

For gist details, there were no significant differences between groups, no significant differences between days, and no significant interaction (Table 2, Repeated Measures ANOVA, F_group_(1,84) = 0.006, *p* = 0.939, (F_day_(1,84) = 0.962, *p* = 0.330, F_day*group_(1,84) = 1.644, *p* = 0.203). For accessory details, there were no significant differences between groups, no significant differences between days, and no significant interaction (Table 2, Repeated Measures ANOVA, F_group_(1,84) = 0.360, *p* = 0.550, F_day_(1,84) = 0.001, *p* = 0.973, F_day*group_(1,84) = 1.089, *p* = 0.300).

### Context recognition task

No significant differences were found between groups for the context recognition task (Table 2, t(93) = −1.32, *p* = 0.19).

### Temporal order task

#### Kendall’s Tau

The Sleep Group showed a significantly lower Kendall’s Tau correlation compared to the Wake Group (Table 2, t(93) = − 2.001, *p* = 0.048). This suggests that the Sleep Group performed better in maintaining the correct sequence of images compared to the Wake Group.

#### Spearman’s rank correlation

The Spearman correlation was significantly lower in the Sleep Group than in the Wake Group, (Table 2, t(93) = − 2.209, *p* = 0.030). A lower correlation indicates a ranking closer to the correct sequence, suggesting that the Sleep Group demonstrated better performance in preserving the correct order of images.

**Table 2:** Memory tasks. Mean scores (± standard deviation) for context recognition, free recall and Temporal order tasks are presented for wake and sleep groups.

	Group			
Tasks	Wake		Sleep	
	Day 1	Day 2	Day 1	Day 2
Free recall (Gist details)	4.45 ± 2.46	4.86 ± 2.48	4.98 ± 1.98	5.10 ± 2.25
Free recall (Accessory details)	7.77 ± 4.07	8.23 ± 3.43	8.10 ± 4.02	7.75 ± 3.43
Context recognition task	--	5.79 ± 1.30	--	5.43 ± 1.37
Temporal order task (Kendall’s)	--	**0.31 ± 0.18**	--	**0.24 ± 0.19**
Temporal order task (Spearman’s)	--	**0.63 ± 0.43**	--	**0.43 ± 0.43**

#### Correlation analysis

We found no significant differences in sleep stage distribution between participants who recognized and those who did not recognize faces in both the TP and TA lineup conditions (see Supplementary Materials, Tables S1 and S2).

**Fig. 4:**
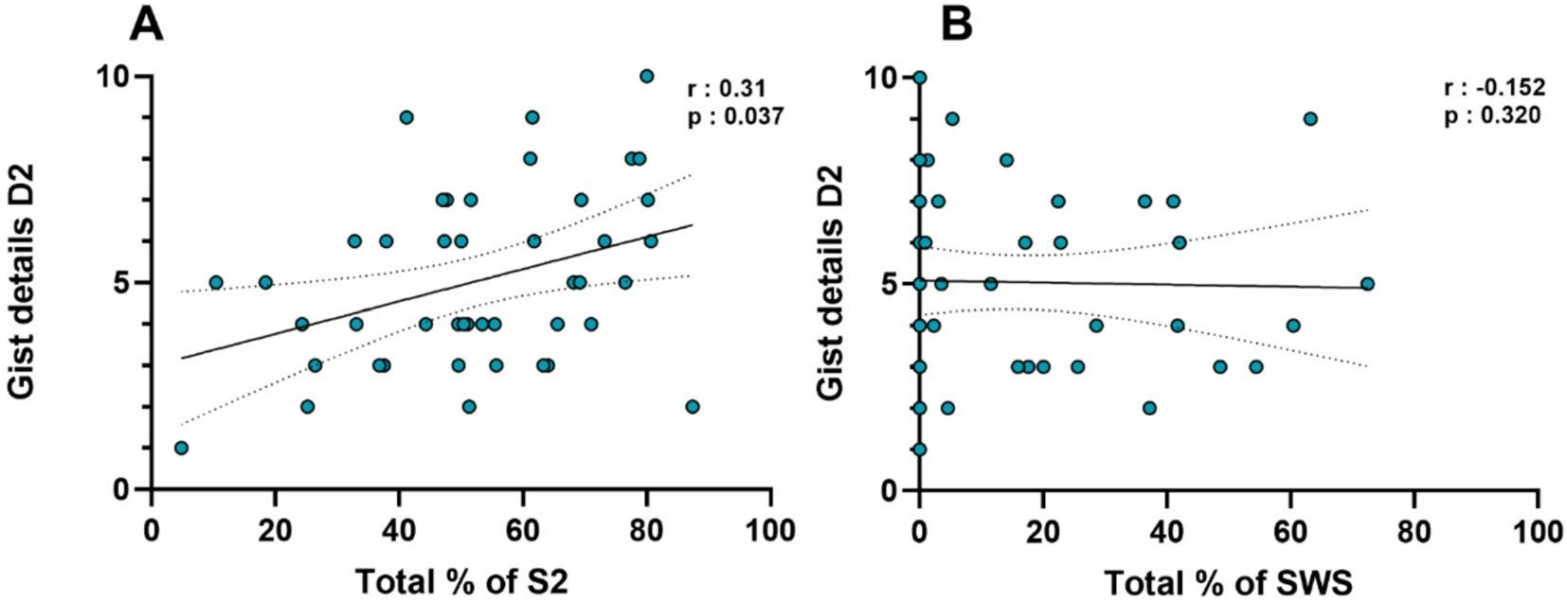
Memory and sleep correlation. **(A)** Correlation between percentage of time spent in S2 and total of gist details on day 2. **(B)** Correlation between percentage of time spent in SWS and total of gist details on day 2.

When examining correlations between time spent in S2 and SWS and memory performance, a higher percentage of time spent in stage 2 sleep was associated with better recall of gist details on Day 2 (r(44) = 0.31, *p* = 0.037). No significant correlation was observed between the percentage of SWS and gist recall (r(44) = − 0.152, *p* = 0.320) (Fig. 4) (see Supplementary Materials, Table S3). No significant correlations were found between the percentage of time spent in SWS, NREM, or S2 and performance on the remaining memory tasks (context recognition and order task) (see Supplementary Materials, Table S4).

#### Symptomatology and sleep quality scales

No significant differences between groups were found regarding the scores on the symptomatology scales or the sleep quality questionnaire (BDI: t(93) = −1.241, *p* = 0.218. GAD-7: (t(93) = 0.132, *p* = 0.895., PSQI: t(93) = −1.25, *p* = 0.213) (Table 3).

**Table 3:** Symptomatology scales and sleep quality. Mean scores (± standard deviation) for anxiety, depression levels, and sleep quality index are presented for wake and sleep groups.

	Group	
Scales and questionnaires	Wake	Sleep
GAD-7	6.52 ± 3.96	6.64 ± 4.56
Depression (BDI)	12.14 ± 8.03	10.28 ± 6.46
Sleep quality (PITTSBURGH)	5.59 ± 2.39	5.04 ± 1.89

## Discussion

In this study, we found that a brief nap before retrieval selectively enhanced eyewitness memory performance by reducing false identifications in target-absent lineups. Importantly, this benefit was not observed in target-present lineups, suggesting that napping may not globally enhance recognition, but rather improve monitoring and decision-making processes critical for rejecting incorrect options when retrieval cues are ambiguous^19,21^. Furthermore, taking into account that all participants had a full night of sleep at home between encoding and retrieval, the core memory consolidation processes were likely comparable across groups^39,40^. The timing of the nap, occurring more than 24 h after encoding, suggests that the nap likely did not interfere with or contribute directly to consolidation, as the window for sleep-dependent memory stabilization was already closed^41,42^. Instead, we attribute the observed improvements to the reduction of homeostatic sleep pressure and the restoration of prefrontal cortex function, particularly those involved in executive control and monitoring processes^17,21^. Importantly, our findings echo those of Stepan et al.^13^, who reported fewer false identifications in target-absent lineups after nocturnal sleep. However, their study involved a full night of sleep without sleep recordings, making it difficult to disentangle consolidation-related effects from retrieval-related improvements. By contrast, our use of a short, NREM-rich nap without REM sleep allowed us to isolate the effect of NREM sleep on retrieval processes. Although no direct correlation between NREM sleep stages and accuracy was found in our sample, the pattern suggests that NREM might contribute by restoring prefrontal functions associated with source monitoring^20,43^. This restoration could enhance the ability to reject incorrect lineup members in the absence of the target. Interestingly, Morgan et al.^14^ did not replicate the post-encoding sleep benefit on lineup performance. One possible explanation for the discrepancy between Stepan et al.^13^ and Morgan et al.^14^ results could be related to the sleep need of the different samples. Sleep-deprived individuals exhibit greater synaptic saturation^16^, leading to impaired prefrontal function and increased false alarms^21^. Thus, in samples with higher baseline sleep need or greater sleep deprivation, the restorative effects of sleep, via downscaling of synaptic strength and enhancement of cortical efficiency, might become more evident.

Furthermore, confidence-accuracy characteristic (CAC) analyses showed that both groups followed the expected exponential relationship, with the sleep group exhibiting a slightly steeper function in the target-absent condition. This pattern indicates that higher confidence responses were generally associated with greater accuracy in both groups, consistent with prior CAC findings^44^. Notably, logistic regression revealed that confidence was a consistent predictor of correct identifications across both conditions, while neither group nor sleep architecture (e.g., SWS or S2) reached significance. Thus, while the nap improved behavioral performance in rejecting absent targets, this effect was not clearly driven by specific sleep stages, at least within the limits of our sample size.

It is important to highlight that the negative results of the ROC curves can be explained by several factors. First, the relatively small sample size may have limited the statistical power to detect differences between groups. Second, the sleep-related benefit emerged specifically in the target-absent condition rather than across all trials, reducing the overall effect size captured by the ROC analysis, which aggregates data across both TP and TA lineups. These factors likely diminished the sensitivity of ROC curves to detect subtle but meaningful improvements in monitoring and decision-making processes following sleep. Complementary analyses, such as chi-square tests and CAC curves, revealed that napping enhanced correct rejections and improved the confidence-accuracy relationship. Together, these findings highlight the importance of using multiple analytical approaches to fully understand the effects of sleep on eyewitness memory performance.

Regarding performance on the other memory retrieval tasks, we found that napping before retrieval did not improve performance on free recall or context recognition. This absence of effects could be attributed to ceiling effects and limited task sensitivity. Both tasks were included to explore whether pre-retrieval sleep could modulate hippocampus-dependent processes beyond face recognition. While prior work has shown benefits of post-learning sleep for free recall and for context recognition^12,40,45^, these effects have largely been observed in consolidation paradigms, and to our knowledge no studies have examined their retrieval. In this context, the absence of effects in our study suggests that a short nap before retrieval may not influence these memory types to the same extent as it affects item recognition in a lineup. In contrast, temporal order memory showed a significant benefit from napping, as evidenced by lower Kendall’s Tau and Spearman’s rank correlation values. This suggests that sleep, even a short nap prior to retrieval, may enhance episodic sequencing. These findings are consistent with the reinstatement hypothesis^46^, which proposes that retrieval success depends on the reactivation of neural patterns engaged during encoding. By reducing cognitive fatigue, the nap may have facilitated more efficient reactivation processes.

While no direct relationship was found between facial recognition performance and time spent in SWS or S2, exploratory analyses revealed a positive correlation between S2 sleep duration and gist recall on day 2. These results open a new perspective on the role of sleep in memory processes. Although the effects of sleep on memory consolidation have been extensively studied^40,41,47^, and to a lesser extent on memory encoding^48,49^, our findings introduce a novel discussion about the role of NREM sleep in memory retrieval. Sleep may enhance retrieval not only through synaptic downscaling, improving neural efficiency, but also by enhancing metacognitive monitoring processes. This improvement could result not only in higher accuracy in learned tasks but also in a better ability to judge the reliability of retrieved memories and make more precise decisions. We hope these findings will pave the way for future research into the contributions of sleep, especially NREM sleep, to memory retrieval mechanisms.

It is important to summarize the limitations of the study. First, the sample size, particularly for sleep-based ROC analyses, may have limited the statistical power to detect group differences. Although our total number of participants was smaller than in most laboratory-based eyewitness ROC studies and substantially smaller than in large-scale online ROC studies (e.g., Wooten et al.^50^, each participant in our study contributed both target-present and target-absent decisions, increasing the number of observations per participant relative to designs where only one lineup decision is collected. Nonetheless, the analyses may still have been underpowered to detect small effects, particularly given the logistical constraints of in-lab polysomnography compared to online data collection. Second, although our behavioral findings support a role for sleep in reducing false identifications, causal inferences about the specific contribution of sleep architecture remain tentative due to the lack of significant regression effects involving sleep parameters. Finally, the generalizability of a short nap in controlled laboratory conditions to real-world forensic procedures requires further investigation. Our findings have important implications for forensic policy and eyewitness protocols. Given that most real-life crimes are encoded under stressful and uncontrolled conditions, interventions targeting the retrieval phase, such as recommending that witnesses take a brief nap prior to visiting the police station for lineup identification or assessing their sleep quality beforehand, may represent a low-cost and effective strategy to reduce wrongful identifications, especially in ambiguous or target-absent situations. While logistically challenging, incorporating rest opportunities or delaying retrieval tasks until after a short nap could enhance eyewitness reliability, particularly in high-stakes contexts.

In summary, a short nap taken before memory retrieval improved recognition accuracy specifically in target-absent lineups, where the risk of false identifications is particularly high. This benefit does not appear to result from enhanced memory consolidation, as both groups had a full night of sleep after encoding. Instead, the improvement is likely attributable to the restorative effects of sleep on attentional control and higher-order cognitive processes involved in retrieval. Importantly, the sleep group exhibited a stronger relationship between confidence and accuracy in the target-absent condition. This finding suggests that sleep may enhance metacognitive monitoring by supporting the ability to evaluate the reliability of retrieved memories. Such improvements in confidence calibration, understood as the degree to which a person’s expressed confidence corresponds to their actual accuracy, are especially relevant in forensic contexts, where overconfident errors can have serious legal consequences.

## Materials and methods

### Participants

103 residents of the Metropolitan Area of Buenos Aires, Argentina participated in the study. They were recruited through advertisements on social networks. Prior to their participation volunteers signed an informed consent and the study was approved by the Comité de Ética Humana (CEH), University of Buenos Aires (UBA). Additionally, all experiments were performed in accordance with relevant guidelines and regulations. Participants reported no history of psychiatric, neurological or sleep disorders, nor taking any medication at the time of the experiments. 8 subjects were excluded from the analysis because they did not sleep (4), did not follow the instructions (1), did not complete all experimental sessions (3). Participants assigned to the nap condition who did not reach stage 2 sleep, as determined by the appearance of the first sleep spindle or K-complex in the polysomnographic recording, were excluded from the analyses rather than reassigned to the wake group. The final sample included 95 participants, with ages ranging from 18 to 40 (22.95 ± 4.43 years). This group distribution meets the minimum of 29 participants per group established in our a priori power analysis for the between-subject Sleep vs. Wake comparison. Excluding “nap-intended but awake” participants from the wake group was based on both methodological and theoretical considerations. Methodologically, reallocating these participants post hoc would have violated random assignment and introduced heterogeneity into the wake group, as the nap condition involved distinct procedural elements (e.g., sleep-conducive environment, EEG setup, expectation of sleeping) absent from the wake protocol. Theoretically, our key contrast of interest was between memory retrieval following actual sleep versus sustained wakefulness. Including “nap-intended but awake” participants in the wake group would have created an intermediate condition that is not equivalent to either, potentially confounding interpretation. This approach is consistent with prior sleep research using similar designs^51^.

### Procedure

The materials used for this experiment were the same as used in Leon et al., 2024. Participants were randomly assigned to either a Sleep group (Age: 22.30 ± 4.68; *n* = 51) or a Wake group (Age: 23.70 ± 4.05; *n* = 44). For the Sleep group, all subjects spent an adaptation day in the sleep lab including placement of electrodes before the experimental day. On the adaptation day, participants arrived at the lab between 12:00 and 17:00 h. First, they signed the informed consent form and thirty minutes later they were prepared for polysomnographic recordings and were then allowed to sleep in a quiet, darkened room. After the short nap volunteers were awakened and electrodes were removed. The adaptation day and the experimental day 1 were separated by at least a week. The experiment was performed from 12:00 h to 17:00 h. On day 1, participants had to complete the Beck Depression Inventory-II^52^, the Generalized Anxiety Disorder 7 (GAD-7)^53^ and the Pittsburgh Sleep Quality Index (PSQI)^54^. After that, they watched a short aversive video (~ 1 min) and recalled the event. On day 2 (24 h later), participants in the Sleep group arrived at the sleep lab, where they were prepared for polysomnographic recordings before taking a short nap of 60 min. After waking up, they waited for 30 min before doing the remaining tasks to prevent sleep inertia. The Wake group followed the same experimental procedure, except for the short nap. Day 2 involved completing two sequential lineups (Target-absent and Target-present lineups). Following this, participants performed another free recall task (same as day 1) and completed a selection and chronological order task. It is important to note that each participant completed both target-present and target-absent lineups, contributing more than one decision to the ROC analysis. This design increases the number of data points per participant compared to typical eyewitness ROC studies in which each participant provides a single lineup decision.

### Sleep recordings

#### Nap duration

We employed a 60-minute nap opportunity, *which resulted in an average total sleep time of approximately 40 min (M = 37.0 min*,* SD = 15.44)*, allowing most participants to progress beyond Stage 1 into Stage 2 and often into SWS. This duration was intentionally selected to maximize the probability of obtaining NREM sleep rich in slow wave activity (SWA; 0.5–4 Hz, encompassing slow oscillations 0.5–1 Hz plus delta 1–4 Hz), while minimizing the likelihood of entering REM sleep. REM sleep typically emerges after ~ 70–90 min from sleep onset in healthy young adults^18^ and is more closely associated with integration and abstraction processes^55,56^, which could confound the interpretation. Importantly, NREM sleep, particularly stages rich in SWA, has been linked to global synaptic downscaling and the restoration of neural efficiency, as proposed by the synaptic homeostasis hypothesis^16,17^. By using a NREM-rich, REM-free nap more than 24 h after encoding, we aimed to harness these homeostatic processes to optimize retrieval-related performance while minimizing contributions from sleep-dependent consolidation.

#### Polysomnography

Standard polysomnographic recordings were obtained using BrainAmp amplifiers (Brain Products), including electroencephalography (EEG), electromyography (EMG), and electrooculography (EOG). Six electrodes were used for EEG, placed at F3, F4, C3, C4, P3 and P4, according to the International 10–20 system, referenced to electrodes attached to the mastoids. Data were recorded at a sampling rate of 250 Hz. EEG data were band-pass filtered offline between 0.16 and 35 Hz, and a notch filter at 50 Hz was applied to remove line noise. Recordings were scored according to standard criteria as wake, stage 1, stage 2, stages 3 and 4 (SWS), and REM sleep^57^. Analysis was based on epochs free of visually identified EEG artifacts.

#### Power spectral analysis

Power density was calculated separately for all sleep epochs of sleep stage 2, SWS (sleep stages 3 plus 4)). Only artifact-free intervals were analyzed by Fast Fourier Transformations (FFT) with an adapted Hanning window applied to subsequent blocks of 2500 data points (∼10 s), with 1250 data points overlap (∼5 s). Individual mean power density was averaged across all electrodes in the following frequency bands: slow oscillations (0.5–1 Hz), delta (1–4 Hz), theta (4–8 Hz), slow spindle (9–12 Hz), fast spindle (12–15 Hz), beta (15–30 Hz), and alpha (8–13 Hz).

### Video

The video presented lasted about one and a half minutes and was filmed from a first-person perspective. It was a staged robbery in a cafe, where two individuals entered with the excuse of needing to use the restroom. The café featured three occupied tables, one of which provided the point of view for the first-person camera. As a result, viewers experienced the scene as if they were an eyewitness. During the robbery, the intruders announced their intentions, each visibly armed with a handgun, ensuring that the potential influence of weapon presence was balanced across perpetrators (weapon focus effect^24^, and demanded money and valuables from both the cash register and the customers. The two perpetrators were visually distinct: one was taller, bald, and had a lighter skin tone; the other was shorter, had wavy hair, and a darker complexion. These differences in height, hairstyle, and skin tone reduced potential facial similarity effects, which can influence eyewitness identification in multi-perpetrator crimes^58^. One thief centered on the employees, a manager and a waitress, while the other moved among the tables. Each thief approached the simulated person (the camera) individually, looking directly at it for three seconds while issuing threats and demanding valuables. This arrangement ensured that all participants had an equal opportunity to observe and encode the facial features of both perpetrators. After collecting the stolen items, the thieves left, leaving behind a tense and distressed atmosphere.

### Lineup

The lineup and lineup fairness was the same as in León et al.^59^. This task was carried out on day 2. The lineups were presented in a simultaneous format, in which all six photographs were displayed together. This choice was based on its frequent use in real forensic practice within Argentina, including in the Province of Buenos Aires. While some jurisdictions abroad debate the relative merits of simultaneous versus sequential formats^37,60^, in our context there is no nationwide regulation mandating one format over the other, and in many provinces simultaneous lineups remain the most common procedure. After entering the experimental room the subjects received the following instruction: ‘’Now you are going to observe a line-up with six suspects, possibly including one of the people who robbed the bar in the video. Take your time to watch them and then if you identify the suspect tell me the number that accompanies his photo. You can choose someone, or tell me that you do not recognize anyone as one of the perpetrators of the robbery’’ and they went through the first lineup. Volunteers could choose one of the options or say that it was not there, without a time limit. Then the procedure was repeated with the second lineup. The order of the lineups (target-present and target-absent) was counterbalanced across participants to control for potential order effects. Participants could receive one of four combinations:

- Target 1 present - target 2 absent.
- Target 2 present - target 1 absent.
- Target 2 absent - target 1 present.
- Target 1 absent - target 2 present.

After each recognition, participants were presented with a confidence scale ranging from 0 to 100, where they indicated their confidence level in their decision. There was no time limit for the subjects to make a decision. If participants chose the perpetrator, the answer was classified as hit (target selection); if they chose a foil or rejected the lineup, miss (foil selection plus incorrect rejection). Images of the suspects were displayed on the computer, on the same monitor that the video was displayed on. All the images were in black and white and both the targets and the fillers had the same posture and neutral facial expression in the photo.

For TP lineups, a “hit” was recorded when the perpetrator was correctly identified; a “miss” was recorded when a foil was selected or the lineup was incorrectly rejected. For TA lineups, a “correct rejection” was recorded when the participant rejected the lineup, and a “false identification” was recorded when any foil was selected. No designated innocent suspect was included in the TA lineups; therefore, following prior studies using this approach (e.g., Mickes et al.^37^, all foil identifications in TA lineups were treated as equivalent to an innocent suspect identification for analysis purposes.

The ROC curves were computed as follows: the true positive rate (TPR; y-axis) was the cumulative proportion of correct culprit identifications in TP lineups at or above each confidence level, calculated as the number of correct identifications in TP lineups divided by the total number of TP lineups. The false positive rate (FPR; x-axis) was the cumulative proportion of foil identifications in TA lineups at or above each confidence level, calculated as the number of foil identifications in TA lineups divided by the total number of TA lineups. Lineup rejections in TA were counted as correct rejections and did not contribute to the FPR. We opted for a full ROC approach because no designated innocent suspect was included in the target-absent (TA) lineups. Following the methodology of Mickes et al.^37^, all foil identifications in TA lineups were treated as equivalent to an innocent suspect identification. This allowed the construction of full ROC curves as cumulative hit versus false-alarm rates.

Lineup fairness was assessed using four groups of participants recruited to assess the fairness of each subject’s alignment test. One lineup included target 1, another target 2, another did not have target 2 while the other lineup did not have target 1 following a 11 mock-witness approach^61^. Simulated witnesses, who had no prior knowledge of the crime video or the identity of the target, were given a brief description of the target and then asked to identify the suspect from a list based on this description. To ensure fairness in the lineup, it was crucial that the simulated witnesses could not identify the suspect at a higher rate than what would be expected by chance (lineup bias). Additionally, their choices should be evenly distributed among the lineup members (lineup size^62^). The Acceptable Lineup Members technique (ALM) developed by Malpass and Devine^61^ was employed to measure the lineup size. A minimum percentage of 75% was considered acceptable as the expected probability^62^. The lineup bias was assessed using the Functional Size method^63^. In the lineup with the target 1, 119 mock witnesses participated, an ALM of 4.50 and a Functional Size of 3.83 were obtained. In the lineup with the target 2, 62 mock witnesses participated, an ALM of 5.79 was obtained and a functional size of 2.81. In the lineup without the target #1, 99 mock witnesses participated and an ALM of 4.57 was recorded. In the lineup without the target 2, 63 mock witnesses participated and an ALM of 5.38 was recorded.

### Free recall

Participants were instructed to provide a verbal free recall of what they had seen and heard in the crime video. This task was performed twice: on day 1, immediately after viewing the video, and on day 8, following the lineup task. All responses were recorded using a voice recorder and subsequently transcribed for analysis. Participants were not given a time limit to complete their testimony.

### Context recognition task

In this task, subjects were shown eight pairs of photos. Each pair corresponded to a scene in the video, but one of the two was altered with a detail added or deleted. The subjects had to decide from each pair of photos which was the correct one. The pairs were displayed on the monitor sequentially.

### Temporal order task

This task began after the context recognition task, since the participants chose the 8 photos that they believed were correct and then they were asked to order them chronologically.

### Symptomatology and sleep scales

Three standardized assessments were administered for control purposes: the Beck Depression Inventory-II (BDI-II), a 21-item multiple-choice questionnaire that measures the severity of depressive symptoms^52^; the Generalized Anxiety Disorder-7 (GAD-7), a screening tool for assessing anxiety levels and their severity^53^; and the Pittsburgh Sleep Quality Index (PSQI), a self-report questionnaire that evaluates sleep quality over the past month^54^.

### Statistical analysis

Statistical analysis was carried out using the IBM software SPSS Statistics 25.

### Facial recognition task analysis

For the lineup, correct answers were defined as identifying the target (hits), and incorrect answers as choosing a filler (false alarms) or saying that the perpetrator is not present (incorrect rejections). In the TA lineup, correct responses were counted when participants selected the option “not present” (correct rejections), while incorrect responses were recorded when any other option was chosen (false alarms). To evaluate performance on the lineup task, receiver operating characteristic (ROC) curves were generated based on participants’ confidence ratings (ranging from 100% to 0%). These curves plotted the cumulative proportion of correct culprit identifications in TP lineups at or above each confidence level = (number of correct identifications in TP lineups) ÷ (total number of TP lineups) against the cumulative proportion of foil identifications in TA lineups at or above each confidence level = (number of foil identifications in TA lineups) ÷ (total number of TA lineups) (following Gronlund et al.^38^. Lineup rejections in TA were counted as correct rejections and did not contribute to the FPR. To statistically assess differences between groups, we computed the area under the curve (AUC) for each group using the pROC package^64^. Higher AUC values reflect a greater ability to distinguish between guilty and innocent suspects. Finally, a z-test was conducted to determine whether the AUCs differed significantly between groups in each experiment.

To evaluate the relationship between the accuracy of the elections and the confidence attributed to them, CAC curves were performed. To calculate the value of the correct proportion corresponding to each confidence level (low 0–50%, medium 50–80%, or high 80–100%) the following formula was used: *# Correct identifications/# Correct identifications + # Incorrect identifications*^44^. Additionally to the CAC curve two separate curves for each condition were made. For the TP condition the formula *# Correct identifications/# Correct identifications + # False alarms + # Incorrect rejections* was used and for the TA condition the formula *# Correct rejections/# Correct rejections + # False alarms.*

Further analyses using chi-square (χ2) tests were conducted to compare the number of hits and misses for each group in both lineup types (TP and TA). In the TP lineup, a hit was defined as correctly identifying the culprit, while a miss was defined as either choosing a filler or incorrectly stating that the culprit was not present. In the TA lineup, a hit corresponded to correctly stating that the culprit was not present, and a miss was defined as choosing any lineup member. These comparisons provided additional insights into the performance of each group across different lineup scenarios.

### Free recall task analysis

To analyze the recall recordings, the key scenes from the video were first identified. These scenes included the central details of the narrative. A total of 12 main details were established, making 12 the highest possible score for central details. These central details represented the gist of each scene.

Additionally, the number of accessory details added to each main scene was quantified. This score did not have a predefined maximum.

Participants’ verbal descriptions in the free recall task were audio-recorded, transcribed, and segmented into information units, defined as discrete ideas or details corresponding to specific events or elements depicted in the target video. To establish the scoring template, three independent researchers watched the video and identified the key scenes that captured the main events of the storyline. Based on their consensus, a final list of scenes was compiled, with each scene corresponding to one information unit. For each participant, a unit was awarded one point if the scene was mentioned and described accurately. Units were scored as correct or incorrect depending on their factual correspondence with the video. Two independent raters, blind to experimental condition, scored all transcripts, and discrepancies were resolved through discussion until agreement was reached.

Since central and accessory details assess distinct aspects of memory and operate on different scales, they were treated as separate variables and analyzed independently. This approach is supported by research indicating that these two types of memories function differently in emotional or stress-related contexts^24,65^. We conducted a repeated-measures ANOVA with “day” as a within-subject factor (two levels: day 1 and day 2) and “group” as a between-subjects factor (two levels: Sleep and Wake).

### Context recognition task

The correct recognitions of images were counted. The maximum number of correct answers was 8, and the minimum was 0. The results between groups were compared using the Student’s t-test.

### Temporal order task

To analyze the differences in image sequencing between groups, we employed two non-parametric indices: Kendall’s tau and Spearman’s rank correlation. Kendall’s tau assesses the concordance between the participants’ sequences and the correct order by evaluating the number of pairwise inversions. Spearman’s rank correlation provides a complementary measure of ordinal association, capturing monotonic relationships between sequences. These indices were chosen as they respect the ordinal nature of the data and allow for a robust comparison of sequencing patterns across groups. The scores of the groups were compared using the Student’s t-test.

### Sleep analysis

For all the sleep analysis sleep stages were defined as follows: S1, S2, S3, S4, REM, NREM (S2, S3 and S4) and Slow Wave Sleep (SWS) (S3 & S4).

### Correlation analyses

Independent t-tests were conducted to assess differences within the sleep group based on performance in the facial recognition task under each condition (present and absent) and to examine variations in sleep stage distribution. Pearson correlation analysis was conducted to examine the relationship between sleep stages and performance on the free recall, context recognition task and the temporal order task.

### Power density analysis

Independent t-tests were conducted to assess differences within the sleep group based on performance in the facial recognition task under each condition (present and absent) and to examine variations in sleep stage distribution.

Pearson correlation analysis was performed to determine the correlation between power density during sleep and memory measures, including free recall, context recognition and temporal order task.

Exploratory analyses were conducted across all sleep stages and electrode sites to examine the remaining frequency bands.

### Symptomatology and sleep scales analysis

Scores of the symptomatological scales (BDI and GAD) and the PSQI (Sleep Quality) were taken as total values (the sum of all test items). For both groups independent means were calculated.

### Statistical power analysis

Analytical approaches were selected to match the structure of the data and to directly test our hypotheses. Mixed-effects logistic regression models were used for lineup decisions to account for the repeated-measures design, modeling both fixed effects (e.g., Sleep/Wake condition, lineup type) and random effects (participants). Chi-square tests were used to compare categorical frequencies for specific decision outcomes. Confidence–accuracy characteristic (CAC) analyses were performed to examine the relationship between confidence and accuracy, following recommendations in the eyewitness identification literature. ROC analyses quantified overall discriminability between target-present and target-absent lineups, with a full ROC approach adopted due to the absence of a designated innocent suspect in the TA lineups, following prior work using similar designs. For the temporal order and context recognition tasks, we employed non-parametric ordinal association measures (Kendall’s tau and Spearman’s rank correlation) and Student’s t-tests, as these are appropriate for ordinal and interval data, respectively, and allow us to test our prediction that pre-retrieval sleep would modulate hippocampus-dependent retrieval processes. Model assumptions were checked prior to interpretation: residual diagnostics showed no major violations of normality or homoscedasticity for linear models, and logistic models met convergence criteria without problematic multicollinearity.

For all studies the following power analysis were obtained using G*Power 3.1.9.7. A priori power analysis was conducted to determine the required sample size for detecting a significant correlation in the Pearson correlation analysis. The analysis was based on a two-tailed test, an alpha level of 0.05, and a desired statistical power of 0.95. Assuming an expected correlation of *r* = 0.60, the analysis indicated that a minimum sample size of 30 participants was required to achieve adequate power. The critical correlation values were *r*= 0.361. The a priori power analysis considered that each participant would contribute two lineup decisions (one TP and one TA), effectively increasing the number of observations relative to the total N. While our total sample size is smaller than in most laboratory-based ROC studies, and substantially smaller than in recent large-scale online ROC studies (e.g., Wooten et al.^50^), our design ensured sufficient statistical power to detect medium-to-large effects expected based on prior sleep and memory research.

A priori power analysis was conducted to determine the required sample size for detecting a significant effect in a linear multiple regression model. The analysis was based on an F-test with a fixed model assessing R² deviation from zero, using an alpha level of 0.05 and a desired statistical power of 0.80. An effect size of f² = 0.30 and one predictor were assumed. The results indicated that a total sample size of 29 participants (denominator df = 27) was required to achieve adequate power. The critical F-value for this analysis was 4.21, with a noncentrality parameter λ of 8.70. Our final dataset exceeded this threshold, with 51 participants in the Sleep group and 44 in the Wake group, thus meeting the intended power for this main comparison.

Lastly, two a priori power analyses were conducted to determine the required sample sizes for detecting significant associations in the Chi-square tests corresponding to each condition of the study.

For both conditions that were analyzed using a 2 × 2 contingency table, the power analysis was based on a two-tailed test, an alpha level of 0.05, and a desired power of 0.80. We assumed an expected effect size ranging from small to medium (w = 0.30 to 0.50), following Cohen’s conventions. Based on this range, the estimated minimum total sample size required varied from approximately 36 to 88 participants. The critical Chi-square value for this analysis was 3.84, with 1 degree of freedom.

## Supplementary Information

Below is the link to the electronic supplementary material.

Supplementary Material 1

## Data Availability

The datasets used and/or analysed during the current study are available from the corresponding author on reasonable request.
