# Supplementary material for: A nap before retrieval reduces false identifications in target absent lineups
Source: Sci Rep. 2025 Oct 17;15:36385. doi: 10.1038/s41598-025-20471-2 (PMC12534383; doi:10.1038/s41598-025-20471-2)
Supplement: Supplementary file 1 — Supplementary Material 1 [file 41598_2025_20471_MOESM1_ESM.docx]

**Supplementary Material**

**REST TO DISCRIMINATE: NAPPING BEFORE RETRIEVAL REDUCES FALSE IDENTIFICATIONS IN TARGET-ABSENT LINEUPS**

Matías Bonilla, Cristian García Bauza & Cecilia Forcato^#^

**Supplementary Material Overview**

This supplementary material presents additional results from the analyses comparing memory task performance with sleep parameters. The first section focuses on facial recognition performance. We investigated whether responses classified as hits or misses were associated with differences in sleep stage distribution. For each lineup condition, target present and target absent, participants were grouped based on their response type, and independent samples t tests were conducted to compare sleep parameters across these groups.

The second section includes the results of correlation analyses examining the relationship between sleep measures and performance on the remaining memory tasks. Specifically, we assessed whether the percentage of time spent in slow-wave sleep and non-rapid eye movement sleep was associated with outcomes in free recall, context recognition, and temporal order memory tasks.

**Facial Recognition: Independent Samples** *t* **Tests**

**Supplementary table S1**. Summary of independent samples t-tests comparing hit and miss responses in the present condition for sleep stage parameters (S2, SWS, and NREM). Values include t statistic, degrees of freedom, significance level, mean difference, standard error, and 95% confidence intervals.

The following section provides a summary of the results obtained for each condition in the facial recognition task, based on independent samples *t* tests.

| **PRESENT CONDITION** | | | | | | | |
| --- | --- | --- | --- | --- | --- | --- | --- |
| **Independent samples t-test** | | | | | | | |
|  |  | | | | | | |
|  | t | df | Sig. (2-tailed) | Mean Difference | Standard error difference | confidence interval | |
|  |  |  |  |  |  | Inferior | Superior |
| % SPENT IN S2: | -1,072 | 46 | 0,289 | -6,33287 | 5,90830 | -18,22566 | 5,55992 |
| % SPENT IN SWS | 0,897 | 46 | 0,374 | 5,31958 | 5,92921 | -6,61531 | 17,25447 |
| % SPENT IN NREM | -0,083 | 46 | 0,934 | -0,81399 | 9,80708 | -20,55461 | 18,92664 |

| **ABSENT CONDITION** | | | | | | | |
| --- | --- | --- | --- | --- | --- | --- | --- |
| **Independent samples t-test** | | | | | | | |
|  |  | | | | | | |
|  | t | df | Sig. (2-tailed) | Mean Difference | Standard error difference | confidence interval | |
|  |  |  |  |  |  | Inferior | Superior |
| % SPENT IN S2: | -0,739 | 46 | 0,464 | -4,39441 | 5,94643 | -16,36395 | 7,57514 |
| % SPENT IN SWS | -0,347 | 46 | 0,730 | -2,07343 | 5,97305 | -14,09655 | 9,94970 |
| % SPENT IN NREM | -1,282 | 46 | 0,206 | -12,35245 | 9,63723 | -31,75118 | 7,04629 |

**Supplementary table S2**. Summary of independent samples t-tests comparing hit and miss responses in the absent condition for sleep stage parameters (S2, SWS, and NREM). Values include t statistic, degrees of freedom, significance level, mean difference, standard error, and 95% confidence intervals.

**Free Recall: Pearson Correlation Analysis Between Number of Details Remembered and Sleep Parameters**

This section summarizes the results of the free recall task, focusing on the relationship between the number of details remembered and the amount of time spent in slow-wave sleep (SWS), S2 sleep and non-rapid eye movement (NREM) sleep.

|  | | DAY 2 GIST | DAY 2 DETAILS | % SPENT IN S2 | % SPENT IN SWS | % SPENT IN NREM |
| --- | --- | --- | --- | --- | --- | --- |
| DAY 2 GIST | Pearson correlation | 1 | 0,256 | ,311^*^ | -0,152 | 0,074 |
|  | Sig. (2-tailed) |  | 0,090 | 0,037 | 0,320 | 0,627 |
|  | N | 45 | 45 | 45 | 45 | 45 |
| DAY 2 DETAILS | Pearson correlation | 0,256 | 1 | 0,038 | 0,143 | 0,169 |
|  | Sig. (2-tailed) | 0,090 |  | 0,804 | 0,348 | 0,267 |
|  | N | 45 | 45 | 45 | 45 | 45 |
| % SPENT IN S2 | Pearson correlation | ,311^*^ | 0,038 | 1 | -,418^**^ | ,447^**^ |
|  | Sig. (2-tailed) | 0,037 | 0,804 |  | 0,004 | 0,002 |
|  | N | 45 | 45 | 45 | 45 | 45 |
| % SPENT IN SWS | Pearson correlation | -0,152 | 0,143 | -,418^**^ | 1 | ,598^**^ |
|  | Sig. (2-tailed) | 0,320 | 0,348 | 0,004 |  | 0,000 |
|  | N | 45 | 45 | 45 | 45 | 45 |
| % SPENT IN NREM | Pearson correlation | 0,074 | 0,169 | ,447^**^ | ,598^**^ | 1 |
|  | Sig. (2-tailed) | 0,627 | 0,267 | 0,002 | 0,000 |  |
|  | N | 45 | 45 | 45 | 45 | 45 |

**Supplementary table S3**. Pearson correlation coefficients between memory performance on Day 2 (gist and detail scores) and sleep parameters (% time spent in S2, SWS, and NREM sleep). Significant correlations are indicated with p < 0.05 (*) and p < 0.01 (**). The table includes correlation values, significance levels (two-tailed), and sample sizes (N = 45 for all comparisons).

**Context Recognition and Order Task: Pearson Correlation Analysis with Sleep Parameters**

This section presents the results of the context recognition and temporal order tasks. It examines the relationship between memory performance, measured by the number of correctly selected images in the context recognition task and the accuracy scores in the order task, and the amount of time spent in slow wave sleep (SWS). S2 sleep and non rapid eye movement (NREM) sleep.

|  | | CONTEXT RECOGNITION | Kendall Tau | Spearman | % SPENT IN S2 | % SPENT IN SWS | % SPENT IN NREM |
| --- | --- | --- | --- | --- | --- | --- | --- |
| CONTEXT RECOGNITION | Pearson correlation | 1 | -0,055 | -0,032 | -0,012 | 0,130 | 0,129 |
|  | Sig. (2-tailed) |  | 0,711 | 0,829 | 0,933 | 0,377 | 0,382 |
|  | N | 48 | 48 | 48 | 48 | 48 | 48 |
| Kendall Tau | Pearson correlation | -0,055 | 1 | ,979^**^ | 0,013 | 0,169 | 0,156 |
|  | Sig. (2-tailed) | 0,711 |  | 0,000 | 0,930 | 0,252 | 0,289 |
|  | N | 48 | 48 | 48 | 48 | 48 | 48 |
| Spearman | Pearson correlation | -0,032 | ,979^**^ | 1 | -0,014 | 0,104 | 0,071 |
|  | Sig. (2-tailed) | 0,829 | 0,000 |  | 0,927 | 0,481 | 0,632 |
|  | N | 48 | 48 | 48 | 48 | 48 | 48 |
| % SPENT IN S2 | Pearson correlation | -0,012 | 0,013 | -0,014 | 1 | -,439^**^ | ,412^**^ |
|  | Sig. (2-tailed) | 0,933 | 0,930 | 0,927 |  | 0,002 | 0,004 |
|  | N | 48 | 48 | 48 | 48 | 48 | 48 |
| % SPENT IN SWS | Pearson correlation | 0,130 | 0,169 | 0,104 | -,439^**^ | 1 | ,598^**^ |
|  | Sig. (2-tailed) | 0,377 | 0,252 | 0,481 | 0,002 |  | 0,000 |
|  | N | 48 | 48 | 48 | 48 | 48 | 48 |
| % SPENT IN NREM | Pearson correlation | 0,129 | 0,156 | 0,071 | ,412^**^ | ,598^**^ | 1 |
|  | Sig. (2-tailed) | 0,382 | 0,289 | 0,632 | 0,004 | 0,000 |  |
|  | N | 48 | 48 | 48 | 48 | 48 | 48 |

**Supplementary table S4**. Pearson correlation coefficients between context recognition performance (raw scores and rank-based measures: Kendall’s Tau and Spearman’s rho) and sleep parameters (% time spent in S2, SWS, and NREM sleep). Significant correlations are indicated with p < 0.01 (**). The table includes correlation values, two-tailed significance levels, and sample size (N = 48 for all comparisons).
